# Super-resolution mapping of cellular double-strand break resection complexes during homologous recombination

**DOI:** 10.1073/pnas.2021963118

**Published:** 2021-03-11

**Authors:** Donna R. Whelan, Eli Rothenberg

**Affiliations:** ^a^Department of Pharmacy and Biomedical Sciences, La Trobe Institute for Molecular Science, La Trobe University, Bendigo, VIC 3552, Australia;; ^b^Department of Biochemistry and Molecular Pharmacology, Perlmutter Cancer Center, New York University School of Medicine, New York, NY 10016

**Keywords:** DNA repair, homologous recombination, resection, BRCA1, DNA damage

## Abstract

Homologous recombination (HR) is a major pathway for repair of DNA double-strand breaks (DSBs). The initial step that drives the HR process is resection of DNA at the DSB, during which a multitude of nucleases, mediators, and signaling proteins accumulates at the damage foci in a manner that remains elusive. Using single-molecule localization super-resolution (SR) imaging assays, we specifically visualize the spatiotemporal behavior of key mediator and nuclease proteins as they resect DNA at single-ended double-strand breaks (seDSBs) formed at collapsed replication forks. By characterizing these associations, we reveal the in vivo dynamics of resection complexes involved in generating the long single-stranded DNA (ssDNA) overhang prior to homology search. We show that 53BP1, a protein known to antagonize HR, is recruited to seDSB foci during early resection but is spatially separated from repair activities. Contemporaneously, CtBP-interacting protein (CtIP) and MRN (MRE11-RAD51-NBS1) associate with seDSBs, interacting with each other and BRCA1. The HR nucleases EXO1 and DNA2 are also recruited and colocalize with each other and with the repair helicase Bloom syndrome protein (BLM), demonstrating multiple simultaneous resection events. Quantification of replication protein A (RPA) accumulation and ssDNA generation shows that resection is completed 2 to 4 h after break induction. However, both BRCA1 and BLM persist later into HR, demonstrating potential roles in homology search and repair resolution. Furthermore, we show that initial recruitment of BRCA1 and removal of Ku are largely independent of MRE11 exonuclease activity but dependent on MRE11 endonuclease activity. Combined, our observations provide a detailed description of resection during HR repair.

## Significance

DNA resection is an initial, decisive step in the homologous recombination repair pathway of DNA double-strand breaks (DSBs). However, the individual roles and cross-talk of key proteins in this process remain unclear. To resolve the spatiotemporal dynamics of this intricate process, we applied multicolor single-molecule localization microscopy along with robust analytical approaches. Our data reveal the dynamic actions and interactions of MRE11, BRCA1, and CtIP both as a multimer and individually; the recruitment and spatial exclusion of 53BP1; the role of BLM helicase alongside EXO1 and DNA2 nucleases; and the inhibitory mechanisms of MRE11 exo-/endonuclease inhibition. Together, our findings describe important aspects of DNA DSB repair and highlight key differences between repair of clustered damage and sporadic endogenous breaks.

Endogenous levels of genomic stress and damage occur as a result of the unavoidable competition for DNA access from the various DNA-binding proteins responsible for replication, transcription, and epigenetic and topographic modification (1, 2). Even in an ideally functioning replicating vertebrate cell, this results in a handful of double-strand breaks (DSBs) per day (3, 4), which if misrepaired, can lead to cell death or mutagenesis (2, 5, 6). In replicating cells, several interacting, partially redundant processes exist for DSB repair, with two principal repair pathways described: homologous recombination (HR) and nonhomologous end joining (NHEJ) (7, 8). NHEJ is a comparatively straightforward process that involves the direct ligation of two blunt DNA ends (ostensibly the two ends of the DSB), whereas the HR repair process uses a homologous genetic sequence as a template for repair, providing improved fidelity over NHEJ (2, 8). Importantly, HR is specialized for the repair of endogenous DSBs during replication, most commonly caused by replication fork (RF) lesions resulting in characteristic single-ended double-strand breaks (seDSBs) without a coinciding second blunt end and that, therefore, cannot be repaired via NHEJ (9–13). Many of the isolated HR repair steps have been identified and characterized, allowing an overall model of the pathway to be hypothesized (10, 14, 15). After a DSB is generated, ataxia telangiectasia and Rad3 related and ATM serine/threonine kinase orchestrate a DNA damage response signaling cascade that spreads throughout the cell, altering various genomic and metabolic processes (16). At the break, first responder proteins identify the end and mediate pathway choice, although the intricacies of how this is carried out remain unresolved (7, 8, 17–19). Central to the HR repair pathway is the resection process, as orchestrated by the MRE11 nuclease and several cofactors, ultimately committing the break to HR-dependent repair (20). After enough replication protein A (RPA)-coated single-stranded DNA (ssDNA) is generated, the RAD51 recombinase is recruited to form a nucleoprotein filament that performs homology search and invasion for templated repair (21). Repair is then finalized by polymerase-driven synthesis to form double-stranded DNA (dsDNA). Following this, the RF can be reassembled and the D loop resolved, or a converging fork can be ligated creating a double Holliday junction, which can result in either cross-over or noncross-over products (22).

Our present understanding of the complex spatial and temporal organization of HR repair of seDSBs relies on the accumulation and convergence of data generated using an array of biochemical, cellular, and molecular biology techniques. Fluorescence microscopy has been a stalwart feature of these studies, allowing for visualization of the comings and goings of tagged proteins of interest as well as determination of their association with each other at repair sites in vivo (23–25). However, conventional microscopy is restricted by the diffraction of light, which limits colocalization assessments to interspecies distances that could, in reality, be separated by several hundred nanometers (26). Perhaps even more inhibitory to these experiments, conventional fluorescence imaging of cellular DNA repair relies on thresholding methods to identify the brighter and more concentrated protein “foci” (24). In order to be detected above the background level of dispersed proteins, these bright nuclear foci consist of tens, if not hundreds, of fluorophores labeling clusters of proteins (24, 27). For this reason, the vast majority of imaging studies of DSB repair have used either laser or ionizing radiation to induce clustered damage sites (28, 29). These techniques also damage the surrounding cellular environment, generating radical species that can react indiscriminately with proteins and lipids, as well as cause other types of DNA damage in close proximity to any DSBs generated (23, 25). As such, although foci studies have generated important insights into macroscopic DNA repair processes, they may not provide a full description of repair at the comparatively lower level of unclustered endogenous damage, including the relationship that these breaks have with the genetic deficiencies in repair pathways that lead to many cancers (30) as well as neurodegenerative and other aging-related diseases (31).

Previously, we established powerful assays that combined single-molecule super-resolution (SR) imaging with pulse labeling of DNA to visualize the recruitment and localization of DNA damage response proteins to DNA DSBs (27, 32–34). Using this approach, we were able to describe the extended timeline of HR repair at seDSBs as well as the specific roles and cross-talk of key HR proteins including BRCA2, RAD51, and RAD52 (27). In the current study, we focus on the crucial initial processes associated with repair of collapsed RFs, in particular orchestration of resection to produce a long RPA-coated ssDNA 3′ overhang. The combination of replication-specific damage induction, labeling assays, and multicolor single-molecule SR approach engenders a 10-fold improvement in image resolution over conventional fluorescence microscopy. It is also advantageous as it gives all fluorophores detected in the sample equal weighting regardless of their propensity to form clusters or foci under the experimental conditions (26). This contrasts with conventional imaging, which has a finite dynamic range and typically fails to detect individual fluorophore signals due to their very low photon count per pixel compared with the strong pixel reading from a concentrated cluster. With all single-fluorophore emissions being approximately equal and with only one fluorophore emitting onto any individual pixel at any one time, these contrast issues do not occur in single-molecule imaging. Together, these advantages enable examination of individual seDSB sites in vivo and quantification of the spatial and temporal relationships between various HR proteins within the damage foci (27, 35, 36) and comprehensively describe the interplay and interactions of key resection proteins including the nucleases MRE11, EXO1, and DNA2; the damage response helicase Bloom syndrome protein (BLM); and cofactors BRCA1 and CtBP-interacting protein (CtIP).

## Results

### SR Microscopy Analyses Reveal the Spatiotemporal Progression of Individual Repair Foci.

**Fig. 1.**
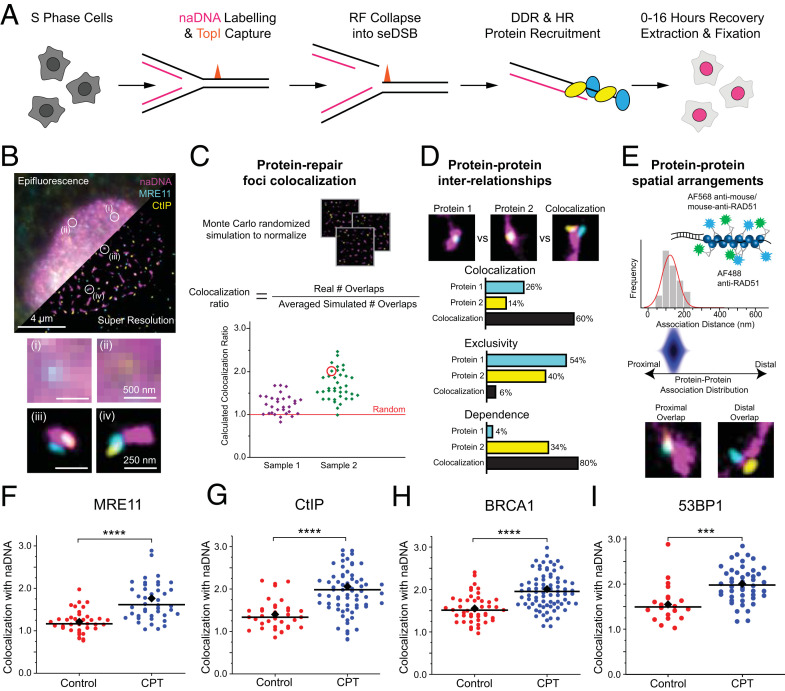
SR imaging of repair proteins recruited to individual seDSBs in human cells. (*A*) The experimental protocol used to label and break RFs into seDSBs in order to capture snapshots of the arrivals, accumulations, and departures of repair proteins. TopI, Topoisomerase I; DDR, DNA damage response. (*B*) Representative epifluorescence (upper left) and SR (lower right) images of a single nucleus damaged and labeled for naDNA (using EdU; magenta), MRE11 (cyan), and CtIP (yellow). (*B*, *i* and *ii*) Representative zoomed-in epifluorescence and (*B*, *iii* and *iv*) SR foci are shown. (Scale bars: *i* and *ii*, 500 nm; *iii* and *iv*, 250 nm.) (*C*) Schematic showing the analytical process for quantifying protein colocalization with repair foci by generating Monte Carlo randomized simulation images for each SR nucleus image and then using the number of overlaps in the random simulations to normalize the real data. For each cell, this normalized “colocalization ratio” can be plotted (e.g., one normalized cell value is circled in red) and compared with truly random levels (=1) and with control levels in untreated control cells. (*D*) The interrelationships between proteins localized to repair foci were further quantified by calculating the percentage of naDNA foci positive for one or both stained proteins. This determined the degree of colocalization between proteins at repair sites, as well as exclusion and dependence relationships. (*E*) The spatial relationship of pairs of proteins colocalized at repair foci could be determined by measuring the distance between the protein foci centers of mass and generating a histogram. This was then extrapolated to generate a protein–protein association distribution two-dimensional heat map. By staining RAD51 with two different fluorophores, we could model the expected association distribution for proximally associated proteins for comparison with proteins that are distally associated at repair foci. Representative foci show proximal and distal overlaps as observed in dual-stained RAD51 (proximal; magenta: naDNA, cyan/yellow: RAD51) and in MRE11 (cyan) and CtIP (yellow; distal; magenta: naDNA). (*F*–*H*) Scatterplots showing quantification of colocalization between (*F*) MRE11, (*G*) CtIP, (*H*) BRCA1, and (*I*) 53BP1 with naDNA in untreated control cells and cells treated with 100 nM CPT for 1 h. Black bars depict medians, and diamonds show the means. ***P < 0.001 in a two-sample Student’s *t* test; ****P < 0.0001 in a two-sample Student’s *t* test.

We used a nonlethal dose of camptothecin (CPT) on S-phase synchronized human bone osteosarcoma epithelial cells (U2OS) to induce spatially separated seDSBs similar to those encountered endogenously (Fig. 1*A*) (11, 37). While we expected CPT to induce some level of replication stress resulting in fork slowing and stalling, its specific ability to capture Topoisomerase I cleavage complexes ahead of RFs is known to result in endogenous-like seDSBs via RF collision (11). Moreover, this approach avoids the widespread and varied damage inherent to radiation-based experiments (38). As we have previously described (27), because of the replication-specific nature of these breaks, pulse labeling of nascent DNA (naDNA) using ethynyl deoxyuridine (EdU) incorporation enabled visualization of individual replication domains, a subpopulation of which would also represent damage/repair foci (39). We have previously determined by direct quantification of damage foci and comet assay that treatment of U2OS cells with 100 nM CPT for 1 h causes an approximate quadrupling of the number of DSBs, enabling detection using our SR assays but not inducing any persistent genomic instability (27, 40).

After 1 h of naDNA labeling and damage induction, cells were transferred to fresh medium and allowed to recover for up to 16 h before fixation and fluorescent labeling. This enabled us to assess several time points throughout the repair process of temporally synchronized seDSBs formed at naDNA (seDSB/naDNA foci) by costaining and localizing pairs of foci-associated protein species (Fig. 1*B*). Bromodeoxyuridine (BrdU) was also incorporated into the genomic DNA and visualized without denaturation in order to follow ssDNA generation (24).

To quantify the specific characteristics of seDSB repair complexes within the three-color SR images, we utilized three tiers of analysis (Fig. 1 *C*–*E*) (27). Specifically, to capture seDSB repair at naDNA, the three tiers of our analysis routine describe the following relevant metrics: 1) normalized nonrandom colocalization of proteins with naDNA foci, 2) protein–protein interplay at naDNA foci, and 3) protein–protein spatial distribution at naDNA foci (Fig. 1 *C*–*E*).

The pairwise colocalization of each protein species with naDNA foci was determined by normalizing to both simulated random levels of overlap and the level of overlap detected in undamaged cells (41). To do this, pairs of color channels were thresholded to minimize noise and false-positive localizations before particle analysis to identify discrete clusters in each of the channels. These clusters could indicate an individual underlying protein that had been detected through the presence of one or multiple secondary antibodies, or it could represent multiple copies of the protein separated by less than ∼80 nm. Using the inherent localization precision (∼5 to 10 nm), multicolor mapping errors (∼10 to 20 nm), and combined antibody spacing (∼10 to 20 nm), we calculated that individual proteins that were separated by more than ∼80 nm would be detected as distinct subclusters (or subfoci) that would appear as a single focus in diffraction limited microscopy. To quantify the area or number of overlaps due to random colocalization in the densely populated nucleus, we simulated images of each single-nucleus image using a Monte Carlo algorithm to randomly redistribute clusters of one color within the nuclear region of interest while maintaining the distribution of the second channel as imaged (27, 41). The number of overlaps calculated for each simulated nucleus could accordingly be used to normalize the number of overlaps in each real nucleus using the ratio of average real overlaps to average random overlaps. Thus, each individual nucleus was assigned a ratio of real to simulated random overlaps, which normalized for any cell to cell differences including cluster density, and cluster and cell size. The average ratio from damaged cells could then be further compared with the average ratio from undamaged cells as shown in Fig. 1*C* (41).

Proteins found to colocalize with seDSB foci during repair could then be further interrogated using three-color data to determine pairs of proteins’ interrelationships within the context of repair foci. By quantifying the proportion of protein signals colocalized with seDSB foci independent of each other as well as together as three-color foci, we could determine the propensity for protein–protein colocalization, exclusion, or dependence at damage sites (Fig. 1*D*). Importantly, this analysis excluded any protein signals not colocalized with naDNA foci in order to specifically analyze repair.

Finally, we developed an analysis to extract subdiffraction spatial information from within damage foci. We examined three-color positive foci (naDNA and pairs of immunolabeled protein) to determine whether the protein signals were closely associated with each other (proximal) or spatially separated by some distance greater than could be explained by inherent labeling and imaging errors (∼80 nm; i.e., more distal) (Fig. 1*E*). Because of the underlying heterogeneity of both the foci (e.g., potentially containing many copies of the same protein) and the imaging conditions (e.g., different labeling efficiencies, different foci orientations relative to the imaging plane, steric hindrance), we did not set out to directly quantify distances between overlapping clusters. Instead, we aimed to determine whether particular proteins likely existed in complexes, as has been shown for many of these proteins by previous work (42), or were more dynamic in their individual associations with the damaged DNA and could interact with the repair foci independent of their traditional binding partners. In particular, we set out to interrogate the ability of MRE11, NBS1, CtIP, and BRCA1 to exist at DSBs uncomplexed with each other.

To assess SR images for internal protein–protein spatial arrangement, we individually interrogated three-color foci by taking an intensity cross-section of the two overlapping protein color channels (at an naDNA focus) to determine the centers of mass of each subfocus and based on this, the interspecies distance. These distance values were collated to produce a histogram, which could be approximated using one or two Gaussian functions. As a standard for closely associated or complexed foci, we imaged RAD51 in cells that had been immunostained using two different fluorophores (Alexa Fluors 568 and 488). As expected, based on our estimates of precision and mapping errors, as well as steric labeling issues, this two-color imaging did not produce perfect overlaps, although with centers of mass distances consistently less that ∼100 nm, such overlaps would appear highly colocalized in any diffraction limited image. The peak protein–protein distance for strongly proximal pairs (i.e., dual-stained RAD51) was determined to be <135 nm, while distal distributions were found to be separated by up to 250 to 300 nm. The fitted Gaussians could be extended to a two-dimensional map with an intensity readout by further calculation of perpendicular Gaussian functions to produce the protein–protein association distribution map shown in blue, which is descriptive of the spatial arrangement within the dual-stained RAD51 foci (Fig. 1*E*). In this case, the distribution is inherently proximal, as we have also shown using dual-stained RPA (40). We then used this analysis and the RAD51 distribution as baselines for comparison with other protein pairs to determine whether they are predominantly proximal, distal, or a combination, within individual damage foci (labeled as “complexed distribution” and shown as red overlaid distributions in figures throughout). This analysis determines whether the proteins themselves are distal or proximal relative to each other and does not take into account the underlying naDNA distribution. Together, these analytical approaches allowed us to define the spatial and temporal behavior of key HR proteins during break recognition and resection.

### 53BP1 Localizes with seDSBs alongside Canonical HR Nucleases and Cofactors during Early Repair.

A protein multimer comprising BRCA1, CtIP, and MRE11 (as part of the MRN [MRE11-RAD51-NBS1] complex) orchestrates resection of DSBs in concert with various other nucleases, helicases, and cofactors (43, 44). The colocalization and interaction of BRCA1, CtIP, and MRN are cell cycle dependent, and the individual proteins are critical for successful HR repair (42, 43). MRE11 is specifically responsible for initiating resection via 5′–3′ endonuclease activity prior to long-range resection by EXO1 and/or DNA2 (45). BRCA1–CtIP interaction at DSBs has also been implicated as critical for HR-dependent repair of replication-associated DSBs but not for restriction endonuclease-induced DSBs (46). Therefore, we first assessed the colocalization of these three key proteins—MRE11, CtIP, and BRCA1—with naDNA foci in both undamaged and CPT-damaged cells (Fig. 1 *F*–*H*). For all three proteins, we detected colocalization levels slightly above random even without damage. We hypothesize that this was due to transient DNA–protein interactions caused by the DNA-binding behavior that these proteins intrinsically possess or due to undamaged levels of transient fork reversals (42, 47). Following 1 h of CPT treatment, we detected a significant increase in MRE11, BRCA1, and CtIP colocalization with naDNA foci as compared with control cells (Fig. 1 *F*–*H*). These data demonstrate the ability of the SR imaging and analysis assay to detect HR repair events even for low doses of CPT and minimal DSB induction levels, as well as comparatively high levels of baseline biological “noise” in the form of associations irrelevant to exogenously induced repair processes. Furthermore, it confirms the fast localization of these key resection-mediating proteins to seDSB sites.

Next, we evaluated the localization and interplay of the HR-inhibitory protein 53BP1. It has previously been shown that 53BP1 recruitment to ionizing radiation-induced damage foci during growth 1 phase (G1) supports the NHEJ pathway choice because 53BP1 can exclude BRCA1 from damage foci (48). However, the interplay between these two proteins during S phase is less clear, particularly because in the absence of BRCA1, 53BP1 is thought to retain its NHEJ-mediating activity regardless of cell cycle phase (49, 50). Furthermore, 53BP1 foci formation has been confirmed throughout the cell cycle regardless of the acting repair pathway, and thus, a 53BP1 binding partner, RIF1, has been identified as key in pathway choice (51). More recently, structured illumination microscopy studies showed that BRCA1 foci formation during S phase caused 53BP1 redistribution to the BRCA1 focus periphery (52). We note that these previous studies have primarily examined radiation-induced clustered damage and that the conclusions of these studies may not necessarily be reflected in DNA damage response to RF-associated seDSBs. In the case of individual seDSBs, we were surprised to detect a comparable increase in 53BP1 colocalization as observed for the aforementioned canonical HR proteins (Fig. 1*I*). This demonstrated that despite its HR-inhibitory function, 53BP1 was still recruited to seDSBs that would eventually be repaired by HR.

**Fig. 2.**
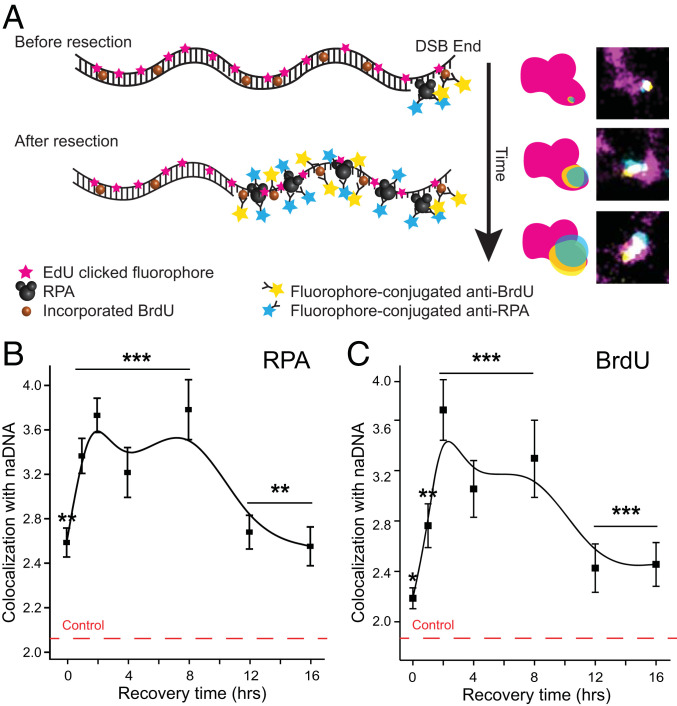
Quantification of RPA and BrdU overlap signals with naDNA shows resection progression. (*A*) A schematic model showing labeling for RPA and ssDNA as resection progresses. (*B*) Kinetic trace of RPA colocalization with naDNA 0 to 16 h after 1 h of 100 nM CPT treatment. (*C*) Kinetic trace of ssDNA (detected as undenatured BrdU) colocalization with naDNA 0 to 16 h after 1 h of 100 nM CPT treatment. SEs are shown. *P < 0.05 based on two-sample Student’s *t* test comparing damaged time point data with control undamaged cell colocalization levels; **P < 0.01 based on two-sample Student’s *t* test comparing damaged time point data with control undamaged cell colocalization levels; ***P < 0.001 based on two-sample Student’s *t* test comparing damaged time point data with control undamaged cell colocalization levels.

To better understand these data, we next set out to determine the temporal progression of these proteins associations with seDSBs throughout HR and specifically, during resection. By immunolabeling RPA and incorporated BrdU, we were able to directly characterize the temporal progression of resected ssDNA generation. For our purposes, BrdU was detected without any denaturation, as would usually be necessary to expose the BrdU epitope in dsDNA (Fig. 2*A*). Areas of colocalization of these immunolabel signals with naDNA were then calculated at seven time points spanning 16-h recovery from CPT treatment in order to determine the kinetics of resection (Fig. 2 *B* and *C*).

This revealed that resection commences quickly following DSB generation, with both ssDNA and RPA accumulating steadily for the first 2 h of recovery before plateauing for a further 6 h. This plateau is indicative of persistent ssDNA coated with RPA. Together, these data establish that resection is predominantly completed during the first 2 to 4 h of repair and that the resulting ssDNA is maintained during homology search and strand invasion steps of HR until ∼8 h of total repair time. After 8 h of the repair process, we detect significant decreases in RPA and ssDNA signals, likely indicating successful synthesis by repair polymerases of the complementary strand to produce dsDNA. Interestingly, the amount of ssDNA does not decrease fully to control levels during the 16 h of observation. Indeed, more than twice as much RPA and ssDNA is detected associated with seDSB foci compared with random levels, similar to those seen in undamaged cells fixed immediately following naDNA labeling. This can potentially be explained by the presence of ssDNA at undamaged replication structures, which may have to be maintained until a converging fork can fully resolve the break site into native duplicated dsDNA (53). It may also be due to persistent seDSBs, which have failed to repair during the course of our observations.

**Fig. 3.**
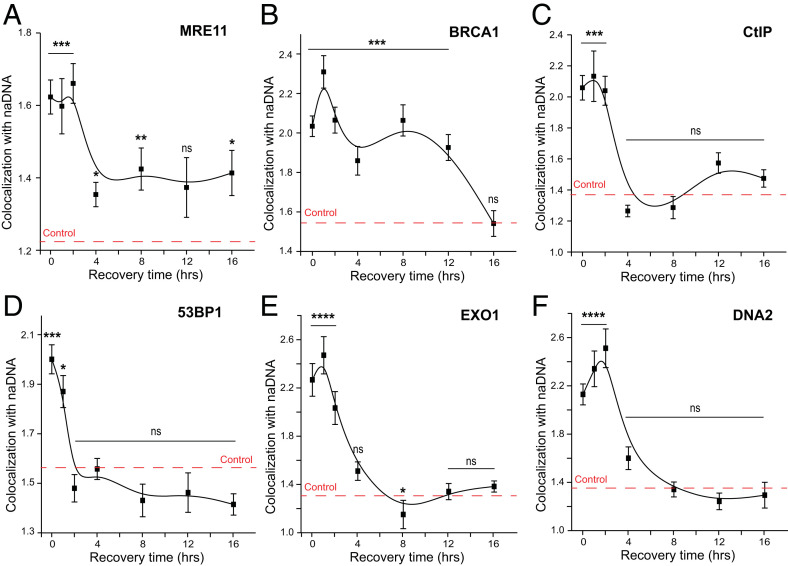
MRN, BRCA1, and CtIP associate with DSBs to orchestrate resection during the first 2 h of repair. (*A*) Kinetic trace of MRE11 colocalization with naDNA 0 to 16 h after 1 h of 100 nM CPT treatment. (*B*) Kinetic trace of BRCA1 colocalization with naDNA 0 to 16 h after 1 h of 100 nM CPT treatment. (*C*) Kinetic trace of CtIP colocalization with naDNA 0 to 16 h after 1 h of 100 nM CPT treatment. (*D*) Kinetic trace of 53BP1 colocalization with naDNA 0 to 16 h after 1 h of 100 nM CPT treatment. (*E*) Kinetic trace of EXO1 colocalization with naDNA 0 to 16 h after 1 h of 100 nM CPT treatment. (*F*) Kinetic trace of DNA2 colocalization with naDNA 0 to 16 h after 1 h of 100 nM CPT treatment. SEs are shown. ns shows *P* > 0.05 based on two-sample Student’s *t* test against undamaged cell colocalization levels. *P < 0.05 based on two-sample Student’s *t* test against undamaged cell colocalization levels; **P < 0.01 based on two-sample Student’s *t* test against undamaged cell colocalization levels; ***P < 0.001 based on two-sample Student’s *t* test against undamaged cell colocalization levels; ****P < 0.0001 based on two-sample Student’s *t* test against undamaged cell colocalization levels.

Having established that resection progresses throughout the first 2 to 4 h of repair, we focused on undercovering the dynamics of early HR proteins during ssDNA generation (Fig. 3). In agreement with their early roles in resection, we found that MRE11, BRCA1, and CtIP are associated most strongly 0 to 2 h after release from CPT. Both MRE11 and CtIP levels decreased to minimally elevated or control levels, respectively, by 4 h (Fig. 3 *A* and *C*). This observation is consistent with the previously reported duration of the resection process (54) as well as the kinetics we detected. BRCA1 persisted at DSBs much longer than MRE11 and CtIP, with colocalization levels at 4 to 12 h comparable with 0 and 2 h (Fig. 3*B*). We hypothesize that this is due to the later HR functions of BRCA1 as an upstream mediator of BRCA2, which facilitates homology search and strand invasion (27, 55).

Temporal mapping of 53BP1 recruitment and residence at damage foci revealed that it strongly associated immediately following CPT damage and 1 h into repair but that it did not persist as long as the examined resection nucleases and cofactors (Fig. 3*D*). Instead, 53BP1 colocalization dropped to control levels by 2 h. This observation is potentially indicative of a mechanism that completely removes 53BP1 from the damage foci by this time. However, removal does not seem necessary for the initial recruitment of HR proteins and the commencement of resection.

To further extract the HR timeline, we next examined EXO1 and DNA2, two 5′–3′ exonucleases hypothesized to work in concert with MRE11 nicking to resect long tracts of DNA at DSBs. Despite a high level of redundancy between the two nucleases and ongoing debate regarding which might act preferentially in vivo, both were observed to be recruited at similar levels (∼2.6-fold above random) in response to seDSB induction (Fig. 3 *E* and *F*). Similar amounts of colocalization were detected immediately following damage and at 1 and 2 h into repair, before returning to control levels at 4 h. Together, these data indicate that resection takes place during the first 2 to 4 h of HR, initially in the presence of 53BP1, and that BRCA1 is the only protein of those we examined with a later role in HR at the DSB site.

### 53BP1 Is Excluded from the DSB, While MRE11, CtIP, and BRCA1 Act Both in Complex and Individually during Resection.

**Fig. 4.**
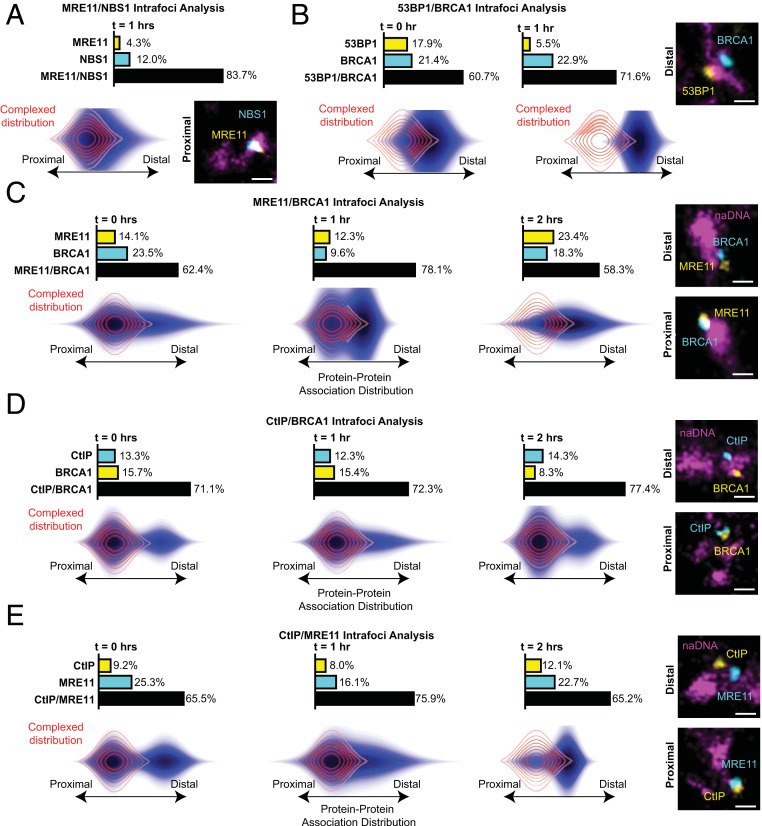
The spatial organization of NBS1, 53BP1, MRE11, BRCA1, and CtIP demonstrates high levels of colocalization at the same repair foci with dynamic spatially proximal and distal arrangements. (*A*) Analysis of the internal organization of MRE11 and NBS1 costained and colocalized with naDNA after 1 h of 100 nM CPT treatment. MRE11/NBS1 shows a high level of colocalization at repair foci with a minor fraction (12.0%) MRE11 only (cumulative bar graph). Colocalized MRE11 and NBS1 have a strongly proximal association distribution at 0 h, indicative of closely associated proteins. (*B*) Analysis of the internal organization of 53BP1 and BRCA1 costained and colocalized with naDNA at 0 and 1 h after 1 h of 100 nM CPT treatment; 53BP1/BRCA1 shows a high level of colocalization at repair foci with minor fractions (5 to 25%) demonstrably MRE11-only or 53BP1-only foci (cumulative bar graph). Colocalized 53BP1 and BRCA1 have a moderately distal association distribution at 0 h that increases in separation at 1 h. (*C*) Analysis of the internal organization of MRE11 and BRCA1 costained and colocalized with naDNA at 0, 1, and 2 h after 1 h of 100 nM CPT treatment. MRE11/BRCA1 shows a high level of colocalization at repair foci with minor fractions (9 to 24%) demonstrably MRE11-only or BRCA1-only foci (cumulative bar graph). Colocalized MRE11 and BRCA1 have a predominantly proximal association distribution at 0 h, both proximal and distal subpopulations at 1 h, and a predominantly distal distribution by 2 h. (*D*) Analysis of the internal organization of CtIP and BRCA1 costained and colocalized with naDNA at 0, 1, and 2 h after 1 h of 100 nM CPT treatment. BRCA1/CtIP shows a similarly high degree of colocalization across time points and even smaller fractions (<8 to 16%) as CtIP-only and BRCA1-only foci. Colocalized CtIP and BRCA1 are predominantly proximal in their association distribution across all times with a persistent population of distal, uncomplexed species. (*E*) Analysis of the internal organization of MRE11 and CtIP costained and colocalized with naDNA at 0, 1, and 2 h after 1 h of 100 nM CPT treatment. CtIP/MRE11 also shows a high degree of colocalization and minor fractions (8 to 26%) of CtIP-only and MRE11-only foci. Colocalized CtIP and MRE11 initially displayed almost equal fractions in distal and proximal arrangements before a shift toward a more proximal distribution at 1 h and then, toward a predominantly distal distribution at 2 h. In all images, the overlaid red contour map outline shows the modeled distribution of closely associated proteins. Representative distal and proximal foci are also shown. (Scale bars: 250 nm.)

To quantify the organization of these proteins within individual seDSB foci, we used three-color SR foci analysis to determine the protein–protein interrelationships and spatial arrangement (Fig. 1 *D* and *E*). Protein–protein association distribution maps were compared with the dual-labeled RAD51 data (depicted as the red complexed distribution map shown throughout Fig. 4). At the core of DNA resection, the MRN trimer consists of MRE11-RAD50-NBS1. To test the hypothesis that the complex exists stably at the DSB site and that our protein–protein association distribution analysis could be extended beyond dual labeling of a single target, we immunolabeled MRE11 and NBS1 (Fig. 4*A*). The protein signals were found to strongly colocalize (82.7%) with a proximal distribution similar to that observed for the dual-labeled RAD51. The small percentage of NBS1 colocalized with naDNA in the absence of MRE11 (12.0%) could be indicative of nonspecific antibody labeling or of an MRE11-independent but replication-associated pathway. Furthermore, the slight extension of the NBS1-MRE11 distribution toward a more distal arrangement of MRE11 relative to NBS1 indicates some amount of NBS1 or MRE11 acting outside of the MRN complex at these breaks. We hypothesize that this could potentially be due to individual MRE11 or NBS1 proteins being recruited to repair foci prior to complexing. Alternatively, this signal could also be due to uncomplexed NBS1, which has been shown to act independent of the MRN complex within the DNA damage response in a transcriptional silencing pathway (56, 57). The predominately proximal colocalization of NBS1 and MRE11 is, nonetheless, a convincing demonstration that the MRN complex is the dominant arrangement of both MRE11 and NBS1 at seDSBs.

In contrast, although 53BP1 and BRCA1 were found to colocalize well at both 0 and 1 h (60.7 and 71.6%, respectively)—which is to say their signals overlapped within individual damage foci—the spatial arrangement of the proteins shows a distinct difference when compared with the dual-labeled RAD51 or the MRE11-NBS1 (Fig. 4 *A* and *B*). At 0 h, the internal arrangement of 53BP1/BRCA1 foci was found to have very little proximal signal, instead depicting spatial separation of the two proteins. One hour following damage, this separation had increased to display the most significant degree of separation observed in this study. This spatial separation of 53BP1 and BRCA1 is in good agreement with previous reports of 53BP1 being moved to the periphery of repair foci (58, 59). However, because of the different damage induction method employed in this previous work and the single-ended, individual nature of the DSBs we observed, we did not detect 53BP1 encompassing the break but rather repositioned in a subfocus nearby. Such redistribution is likely key in progression of the repair pathway, albeit unnecessary for the initial resection steps to take place. Indeed, we detect significant colocalization of MRE11, NBS1, CtIP, and BRCA1, as well as ssDNA generation and RPA binding, contemporaneous with 53BP1 colocalization at 0 h but prior to the most significant redistribution of 53BP1 seen at 1 h.

The protein–protein interrelationship quantification (Fig. 4 *C*–*E*) of MRE11, CtIP, and BRCA1 confirms previous observations that these proteins strongly colocalize with each other at repair foci with only minor fractions of any protein found to associate without the other protein stained in each pairwise assessment. However, the protein–protein association distribution maps of MRE11, CtIP, and BRCA1 reveal that even though the proteins often co-occupy the same focus, in some individual foci they are proximal, indicating that they are potentially complexed, and at other foci, they are distally related and acting independent of each other. That is to say that across a population of foci taken from many cells, some foci displayed protein signals indicative of close association, thus likely interacting with each other, while other foci displayed protein signals indicative of spatial separation. These distinct subpopulations persisted across all protein pairs for the duration of resection. The detection of these proteins individually at seDSBs shows that MRN, BRCA1, and CtIP are frequently and independently recruited to DSB DNA as single species that may or may not go on to become complexed. Indeed, while CtIP and BRCA1 are initially observed to associate closely with MRE11, after 2 h of recovery, they are exclusively positioned distally to MRE11, although they continue to associate with each other. This is not unexpected given the HR-related activities that these individual proteins exhibit in vitro (43) and the nonessential nature of their interaction (47); however, it contrasts the typically complexed interactions described by current HR models.

### EXO1 and DNA2 Both Combine with BLM to Carry Out Long-Range Resection In Vivo.

Coinciding with resection initiation and orchestration by MRN, we observe EXO1 and DNA2 colocalization, presumably to undertake the longer-range 5′–3′ resection (Fig. 3 *E* and *F*). These nucleases, known to have redundancy in both yeast and human cells, have both been shown to carry out extensive resection in vitro (60), with DNA2 displaying both nuclease and helicase activity, whereas EXO1 displays only nuclease functionality. Previous studies have shown the faciliatory effects of both MRN and BLM on both nucleases (60); however, it remains unknown whether one of these helicases acts preferentially in vivo, especially at low levels of DNA damage induction.

**Fig. 5.**
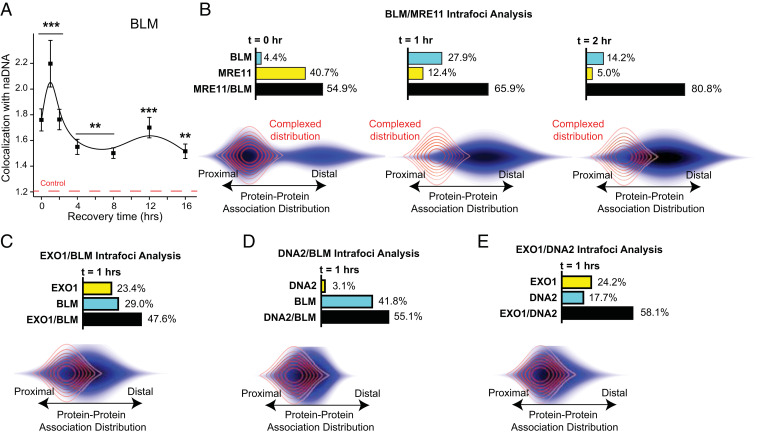
BLM is involved dynamically with nucleases EXO1, DNA2, and MRE11 during resection. (*A*) Kinetic trace of BLM colocalization with naDNA 0 to 16 h after 1 h of 100 nM CPT treatment. **P < 0.01 based on two-sample Student’s *t* test against undamaged cell colocalization levels; ***P < 0.001 based on two-sample Student’s *t* test against undamaged cell colocalization levels. (*B*) Analysis of the internal organization of MRE11 and BLM costained and colocalized with naDNA 0, 1, and 2 h after 1 h of 100 nM CPT treatment. MRE11/BLM shows a moderate, increasing to high level of colocalization at repair foci, with significant MRE11 only observed at the 0-h time point. Colocalized MRE11 and BLM have a mixed proximal and distal arrangement immediately following damage, indicating both complexed and spatially separated species. At 1 and 2 h, the spatial arrangement indicates separation of MRE11 and BLM. (*C*) Analysis of the internal organization of EXO1 and BLM costained and colocalized with naDNA 1 h after treatment with 100 nM CPT for 1 h. Significant levels of EXO1/BLM colocalization at damage foci are detected along with EXO1 and BLM localizations independent of each other. Spatially, colocalized EXO1/BLM shows a moderately distal arrangement indicating the predominance of spatial independence. (*D*) Analysis of the internal organization of DNA2 and BLM costained and colocalized with naDNA 1 h after treatment with 100 nM CPT for 1 h. Significant levels of DNA2/BLM colocalization at damage foci are detected along with BLM localizations independent of DNA2 presence. Minimal DNA2 localization to damage foci is detected independent of BLM, indicating DNA2’s dependence on BLM. Spatially, colocalized DNA2/BLM shows a more proximal arrangement compared with EXO1/BLM, indicating increased interaction, although still with some proteins spatially separated within the foci. (*E*) Analysis of the internal organization of EXO1 and DNA2 costained and colocalized with naDNA 1 h after treatment with 100 nM CPT for 1 h. Significant levels of EXO1/DNA2 colocalization at damage foci are detected along with EXO1 and DNA2 localizations independent of each other. Spatially, colocalized EXO1/DNA2 shows a moderately proximal arrangement similar to DNA2/BLM, indicating some instances of both interaction and spatial separation. SEs are shown.

Furthermore, although BLM has previously been shown to form distinct repair foci upon HR induction, its specific role at seDSBs remains unclear (60–62). Beyond the potential for BLM to be responsible for unwinding DNA in concert with EXO1 and DNA nucleases, it has also been proposed as having stimulatory roles (60). BLM is further hypothesized to facilitate the resolution of D loops formed during invasion and templated DNA synthesis (62). By tracking the kinetics of BLM at naDNA foci, we found that it colocalizes with seDSBs throughout repair (Fig. 5*A*), contrasting with EXO1 and DNA2, which along with CtIP and MRE11, were only detected at 0 to 2 h during resection (Fig. 3 *A*, *C*, *E*, and *F*). Similar to BRCA1, which also has roles in late HR (Fig. 3*B*), this potentially shows BLM’s facilitation of D-loop resolution following strand invasion (Fig. 4*A*) (63). Importantly, BLM, EXO1, and DNA2 all follow similar kinetics during the first 2 h of repair, with EXO1 and DNA2 returning to control levels by the 4-h time point (Fig. 3 *E* and *F*), while BLM drops significantly but not entirely to baseline levels (Fig. 5*A*). This is further evidence of the interplay between these proteins during resection and BLM’s later roles, which appear independent of EXO1 and DNA2 presence.

To further characterize the connection of BLM with early resection complexes, we measured its spatial association with MRE11 (Fig. 5*B*), which revealed a substantial degree of colocalization within repair foci (70.0%) with a smaller fraction of MRE11-only foci (25.0%). The negligible amount of BLM-only foci (5%) indicates that BLM localization to seDSB is dependent on MRN first being recruited and initiating resection. This further indicates that BLM’s role in facilitating long-range EXO1- or DNA2-dependent resection is also dependent on colocalization with MRN at 0 h. At 1 and 2 h, a significant amount of BLM localization to repair foci is observed in the absence of MRE11 (27.9 and 14.2%, respectively), indicating BLM’s ongoing function alongside EXO1 and/or DNA2 without MRE11. Interestingly, the spatial relationship between BLM and MRE11 at seDSB foci is more distal than would be expected if they were tightly coupled throughout the resection process. Immediately following damage, the protein–protein association distribution shows MRE11/BLM in a combination of proximal and distal arrangements. However, at 1- and 2-h recovery, this distribution has shifted to comprise solely spatially separated distal colocalizations. These observations demonstrate that although there is some initial BLM/MRE11 close colocalization, potentially due to interaction, later BLM activities at repair foci are spatially independent of MRE11 and more likely tied to EXO1 and/or DNA2. This is particularly clear from the association distribution maps because during long-range resection, the 5′–3′ directionality of EXO1 and DNA2 necessarily moves them away from the 3′–5′ activities of MRN.

These observations were further confirmed by assessment of EXO1/BLM, DNA2/BLM, and EXO1/DNA2 analyses (Fig. 5 *C*–*E*). EXO1 was found to localize to naDNA foci both alongside BLM and independently (47.6 and 23.4%, respectively), while a significant number of foci stained for EXO1 and BLM only showed the latter protein present (29.0%) (Fig. 5*C*). In contrast, DNA2 localization was found to be dependent on simultaneous BLM recruitment, in line with DNA2’s limited helicase activity (only 3.1% DNA2 recruitment in the absence of BLM) (Fig. 5*D*). However, a significant proportion of foci showed BLM colocalization in the absence of DNA2 (41.8%), likely demonstrating BLM’s ability to stimulate the activities of other nucleases such as EXO1 and MRE11. In contrast to the spatial arrangement observed for BLM/MRE11 and other protein pairs found to be spatially separated (e.g., 53BP1/BRCA1) (Fig. 4*B*), EXO1, DNA2, and BLM were found to be comparatively closely associated (Fig. 5 *C*–*E*). Of the pairs interrogated, EXO1/BLM showed the most distal arrangement, demonstrative of a closer association between BLM and DNA2. Nonetheless, EXO1 and DNA2 were observed to be relatively close to each other when contrasted to the separation of BLM and MRE11. In three-color analysis of EXO1 and DNA2 at naDNA foci, simultaneous colocalization of both EXO1 and DNA2 was found to be the most dominant arrangement (58.1%), although colocalization of only one of the two nucleases was also significant (EXO1: 24.2%, DNA2: 17.7%) (Fig. 5*E*). Collectively, our observations demonstrate that resection is not a single process being undertaken by the MRN-CtIP-BRCA1 multimer but an iterative process that is being performed cooperatively by a number of partially redundant nucleases and their cofactors, with BLM acting dynamically alongside many, if not all, of them (60). Furthermore, BLM was found to predominantly work directly with DNA2, as expected, although simultaneous occupation of individual DSB foci by multiple different nucleases was more common than any singular resection process occurring individually.

**Fig. 6.**
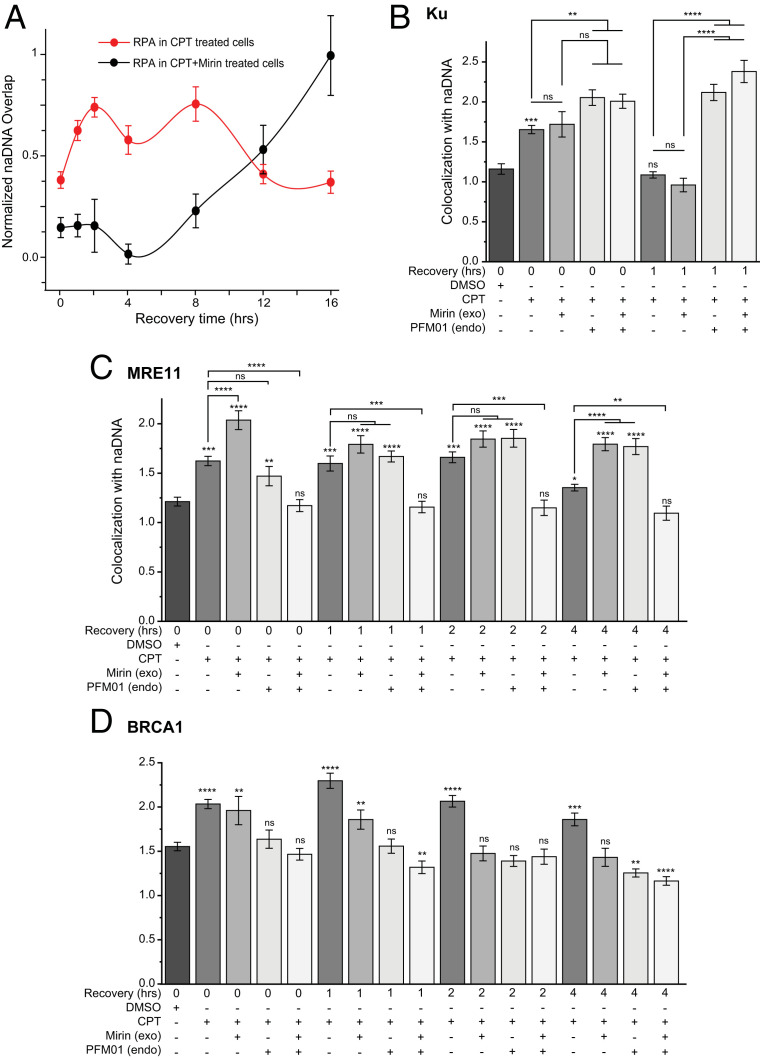
MRE11 nuclease inhibition using Mirin and PFM01 completely abrogates resection at DSBs, causing delayed repair and apoptosis even after removal of Mirin. (*A*) Kinetic trace of RPA colocalization with naDNA 0 to 16 h after 1 h of CPT treatment in combination with Mirin (black trace) or with a DMSO control (red). Adapted with permission from ref. 27, which is licensed under CC BY 4.0. (*B*) Quantification of Ku association with repair foci in control cells, cells treated with CPT, both CPT + Mirin, both CPT + PFM01, and with CPT + Mirin + PFM01. (*C*) Quantification of MRE11 association with repair foci in control cells, cells treated with CPT, both CPT + Mirin, both CPT + PFM01, and with CPT + Mirin + PFM01. (*D*) Quantification of BRCA1 association with repair foci in control cells, cells treated with CPT, both CPT + Mirin, both CPT + PFM01, and with CPT + Mirin + PFM01. Controls are shown for comparison. DMSO signifies dimethyl sulfoxide controls. Student’s *t* test results show significance of difference between CPT-only and CPT + Mirin treatments at indicated times post-CPT. SEs are shown. ns shows *P* > 0.05. *P < 0.05; **P < 0.01; ***P < 0.001; ****P < 0.0001.

To further interrogate the resection process and verify our observations of resection progression in cells, we quantified resection via RPA loading following CPT treatment in the presence of the small molecule drug Mirin, a well-characterized MRE11 endonuclease inhibitor (64). In contrast to the successful resection detected in CPT-only treated cells (red trace in Fig. 6*A*), those exposed to Mirin showed no increase in RPA-coated ssDNA over the first 8 h of CPT recovery (black trace in Fig. 6*A*). At this point, RPA colocalization was observed to steadily increase above RPA association levels detected in CPT-only treated cells, indicating accumulation of an even greater amount of naDNA-associated ssDNA within these cells than in those having undergone resection for HR. We were unable to determine the specific pathway for this RPA accumulation but noted significant loss of cell viability after the 16 h of recovery from CPT/mock treatment in both Mirin and Mirin + CPT-treated cells. We attributed this to the combined stress of the weeklong experiment including cell starvation and many cell cycle and cell signaling effects caused by off-target drug actions and more than 24 h of MRE11 exonuclease inhibition. While we do not aim to interpret these later events, we consider the lack of resection in the Mirin-treated cells a strong indication that the temporal mapping of resection progression in the CPT-only treated cells—and therefore, unperturbed HR repair—is accurate.

To further determine changes to protein recruitment during drug-induced MRE11 inhibition, we selectively inhibited both the endo- and exonuclease activity of MRE11 using PFM01 (20) and Mirin (64), respectively. Previously, Shibata et al. (20) demonstrated that MRE11’s endonuclease activity is upstream of its exonuclease activity and that HR deficiency from endonuclease but not exonuclease inhibition can be rescued by the NHEJ repair pathway. However, the seDSBs generated in our assays preclude repair of this lesion by the NHEJ.

Ku, an NHEJ first responder protein, has been reported as occupying DSBs prior to resection and its removal identified as a potential pathway-determining step (19, 27). In good agreement with these recent observations, we detected Ku at seDSBs immediately following CPT treatment, and by 1 h, we observed that Ku had been fully removed (Fig. 6*B*). Combined CPT + Mirin treatment did not affect either the initial recruitment of Ku nor its removal by 60 min, indicating that although resection is inhibited, neither Ku recruitment nor removal are affected by exonuclease inhibition alone. In contrast, CPT + PFM01 and CPT + Mirin + PFM01 caused increased Ku recruitment and its persistence at the break 60 min following CPT release. This was in good agreement with previous observations of NHEJ repair in PFM01-treated cells and provides further evidence that MRE11 endonuclease activity is required for Ku removal (20).

Surprisingly, in both Mirin- and PFM01-treated cells, significant amounts of MRE11 recruitment were consistently observed and persisted beyond the 2 to 4 h during which resection and most MRE11 removal occurred in cells treated only with CPT (Fig. 6*C*). In the case of exonuclease inhibition (Mirin), increased levels of MRE11 recruitment were detected at 0, 1, 2, and 4 h following damage, despite no resected DNA being produced. With endonuclease inhibition (PFM01), initial MRE11 levels were similar to cells treated only with CPT but still significantly higher than in undamaged cells at 0, 1, and 2 h. After 4 h, in PFM01-treated cells MRE11 association with repair foci remained significantly higher than both control and CPT-only treated cells. Thus, we conclude that neither exo- nor endonuclease inhibition of MRE11 impact its ability to be recruited to DSBs (Fig. 6*C*). However, when both drugs were combined, no significant MRE11 recruitment was observed at any time point. This somewhat counterintuitive observation potentially indicates a combined effect of the endo- and exonuclease activity of MRE11, although we cannot discount signaling inhibition, particularly in light of the lack of potential NHEJ rescue in the presence of blunt seDSBs. It is also very probable that various off-target effects occurred due to the combined effects of cell synchronization and three drug treatments, including dual inhibition of MRE11, which is known to have multiple roles beyond HR (65).

Because MRE11 also plays key roles in DNA damage response signaling and recruiting and stimulating other HR cofactors (66), we next tested the effects of endo- and exonuclease inhibition on BRCA1 recruitment (Fig. 6*D*). However, we note that we could not interpret these observations as due to the drug’s direct effects on resection, on MRE11 itself, or on other signaling. As shown earlier, BRCA1 is recruited to seDSBs immediately after damage induction and persists at the break site throughout HR (Fig. 3*B*). Upon exonuclease inhibition (Mirin), we detect similar levels of BRCA1 recruitment immediately following damage, but by 2 h, BRCA1 levels in Mirin-treated cells have depleted to control levels (Fig. 6*D*). In endonuclease-inhibited cells (PFM01), no recruitment of BRCA1 was detected at any time point; indeed, at 4 h after damage, BRCA1 levels in PFM01-treated cells were significantly lower than even in control cells. Similarly, in cells treated with both Mirin and PFM01, control or lower than control levels were detected at 0 to 4 h after damage. This further supports the determination of Shibata et al. (20) of MRE11’s endonuclease activity as critically upstream of MRE11 exonuclease activity. In agreement, our data show a complete loss of BRCA1 recruitment in cells lacking MRE11 endonuclease ability.

Together, these observations confirm the upstream role of endonuclease MRE11 activity and indicate its potential role in Ku removal and HR repair pathway choice (20, 66). In both nuclease inhibitions, we also demonstrate successful recruitment of MRE11 and high persistence, potentially due to ongoing attempts to initiate repair in the absence of any other potential repair pathway. Furthermore, the perturbations to the HR repair of seDSBs detected upon resection inhibition confirm the spatiotemporal insights into uninhibited HR discerned using the same assays with CPT-only treated cells.

## Discussion

Herein, we have described and applied DNA damage SR imaging assays, which enable the spatiotemporal mapping of repair pathways at the single-molecule, individual DSB level, within a low-damage dose setting. Furthermore, the data presented establish the schedule of key protein arrivals, accumulations, and interactions associated with the resection process of seDSBs formed at collapsed RFs and undergoing HR-dependent repair. Of particular interest is our detection of contemporaneous BRCA1/53BP1 colocalization with repair foci. We show that despite their antagonism of each other and the specifically HR-dependent repair of seDSBs during S phase, 53BP1 remains at the break site for over an hour following damage without abrogating resection or removing BRCA1 (Figs. 1*I* and 3 *B* and *D*). This finding contradicts previous conclusions, drawn predominantly from biochemical data, which highlighted the mutual antagonism and exclusion of BRCA1 and 53BP1 at DSBs (50, 51, 67). Our data are in closer agreement with a more recent study using structured illumination microscopy, also showing BRCA1’s ability to rearrange 53BP1 and chromatin to the periphery of break sites (52). Interestingly, this study described redistribution of the 53BP1 to encircle the damage foci, whereas we see the subfocus of 53BP1 moved to a single punctum nearby the active repair proteins. This likely reflects the use of microirradiation-induced damage in this and many other previous studies, resulting in high levels of clustered DSBs as well as other types of lesions. In contrast, a key advantage of our assays is that we are able to induce and visualize low levels of individual seDSBs that mimic the single-ended nature of endogenous DSBs at RFs (11). Our observations thus better reflect the repair processes of endogenous DSBs and with regard to 53BP1 recruitment, are indeed in agreement with two recent studies that described the interplay of chromatin compaction with 53BP1 association (68, 69). In this previous work, 53BP1 recruitment was shown to be dependent on the chromatin modification H4K20me2, which is diluted following replication (68), and at a megabase scale, over entire γH2AX (phosphorylation of serine-139 H2A histone family X) domains (69). Although these studies highlight the enhanced recruitment of 53BP1 during G1, in order to facilitate NHEJ, they importantly demonstrate the role of chromatin state, and not BRCA1, in mediating 53BP1 accumulation. In light of our findings, we hypothesize that in the case of individual seDSBs (70), rearrangements of 53BP1 and associated chromatin on the macroscale do not occur as they do in clustered damage. Furthermore, our observations demonstrate that 53BP1 colocalization with BRCA1 at the break does not block subsequent successful HR. This calls into question the importance of the physical exclusion of 53BP1 for repair of seDSBs, particularly during early resection steps, and highlights the key role of signaling and RIF1 inhibition in HR pathway choice (71).

To determine the temporal progression of resection, we quantified both ssDNA using BrdU incorporation and immunolabeled RPA over 16 h. This analysis demonstrated that resection was completed during the first 2 to 4 h following seDSB induction and that the resected DNA was then maintained for a further 6 to 10 h during which homology search, strand invasion, and synthesis were carried out prior to eventual competition of repair (Fig. 2) (27). Based on this temporal progression of resection, we were then able to specifically interrogate resection proteins and complexes during the initial 2 to 4 h of repair. We show that BRCA1 localization to the break coincides with the arrival and persistence of its cofactor CtIP and MRE11 as part of the MRN complex, as well as the repair helicase BLM (Figs. 3 and 4*A*) (42, 60). Three-color spatial analysis of MRE11 and NBS1 confirmed that most of these proteins existed in close proximity, likely representing the MRN multimer, but hinted at the possibility of some NBS1 acting individually, potentially in the early stages of MRE11-independent signaling pathways as previously hypothesized (56, 57).

Kinetic analyses of single foci of collapsed RFs revealed the assembly, interactions, and disassembly of resection complexes including MRN, BRCA1, CtIP, and BLM over the course of the first 2 h (Fig. 4). Our direct detection of the varied and contemporaneous presence of these proteins at individual seDSBs demonstrates their dynamic interplay, indicating that both complexed MRN-CtIP-BRCA1 and individual proteins exist and have roles in seDSB repair. Indeed, through spatial analysis we were able to differentiate between potential complexes involving MRN, CtIP, BRCA1, and BLM. During the later stages of resection (2 h after CPT removal), we observed no proximal association between CtIP/BRCA1 and MRN, although CtIP and BRCA1 remained colocalized with each other. This contrasted with early resection, immediately following CPT treatment, when the majority of MRN, CtIP, and BRCA1 were all associated closely with potential for complex formation and activity, although still with a subpopulation of these proteins acting individually. Our data thus demonstrate important roles for these proteins both in various complex configurations and individually, as has been suggested by biochemical data (44, 46, 47, 50, 60). It also indicates potentially different roles for MRN, CtIP, and BRCA1 during early and late resection.

In combination with previous experiments that determined the interactions of the nucleases DNA2 and EXO1 alongside MRE11 and BLM (42, 45, 60), our observations of these proteins colocalizing allow us to infer both nucleases appear to play an active role in resection with no clear preference for one or the other at seDSBs. Although some association between BLM and MRE11 was detected immediately following damage, at 1 and 2 h, these proteins were spatially well separated, indicative of BLM’s role alongside DNA2 and EXO1, which we propose are acting increasingly divergent to any ongoing MRE11 endonuclease activity, recruitment, or signaling. Of DNA2 and EXO1, we observed a high dependence of the former on BLM colocalization within the damage focus and a closer spatial arrangement. Combined with detection of more than half DSBs harboring both nucleases simultaneously, this demonstrates BLM’s direct role with DNA2 and more independent activity by EXO1.

We also detected persistent association of BRCA1 at seDSB foci along with the repair helicase BLM throughout HR, demonstrative of later-stage functions for both these proteins beyond resection (Figs. 3*B* and 5*A*). Combined with our and others’ previous work (27, 40, 72), this observation provides further evidence of a potential role for BRCA1 in facilitating BRCA2 mediation of RAD51 activity during homology search and strand invasion. Similarly, it shows that BLM is responsible for unwinding during resection (60, 73) and most likely acts as a helicase in later repair resolution events (61, 62).

Finally, we further interrogated resection during HR by perturbing the pathway via MRE11 exo- and endonuclease inhibition using Mirin (64) and PFM01 (20), respectively (Fig. 6). Previously, it has been demonstrated that MRE11’s endonuclease activity is upstream of its exonuclease activity. As such, DNA damage induced alongside inhibition of MRE11’s endonuclease but not exonuclease activity can be repaired by the NHEJ pathway because in endonuclease-inhibited cells resection does not commence (20). Pretreatment of cells with Mirin prior to CPT damage resulted in incomplete abrogation of resection as detected by RPA accumulation while also causing an accumulation of MRE11 despite its inability to stimulate resection. Treatment with PFM01 also resulted in some MRE11 recruitment upon CPT-induced damage, although not as strongly as in the Mirin-treated cells. In both cases, MRE11 remained associated with the damage foci 4 h after damage, while in CPT-only treated cells, resection had been successfully undertaken and MRE11 dissociated. Interestingly, a combined treatment of Mirin and PFM01 resulted in no recruitment of MRE11 at any time, likely reflecting the combined lack of any nuclease activity. Supporting the previous findings of potential rescue by NHEJ in response to HR failure, we detected recruitment and persistence of the NHEJ protein Ku in both single-inhibitor and combined treatments. Moreover, we determined that inhibition of exonuclease but not endonuclease activity with Mirin allowed the recruitment of BRCA1, whereas endonuclease inhibition blocked this recruitment, further demonstrating the HR licensing ability of MRE11 endonuclease activity.

Combined, our observations define the complex kinetic interactions between different DSB repair factors at collapsed RFs and highlight the multiple and varied roles of several key HR proteins that are engendered by the dynamic assembly and disassembly of different multimeric complexes.

## Methods

Full methodological details are in *SI Appendix*, *Methods*.

### Cell Preparation.

U2OS cells (American type culture collection: HTB-96) were grown, serum synchronized, and drugged in McCoy’s 5A (Modified) medium. To induce RF stress and seDSB generation, cells were then treated with 100 nM CPT (Abcam; 120115) (11). Cells were preextracted and fixed as described previously (34, 40, 74) immediately following damage or released back into drug-free complete medium for a further 1, 2, 4, 8, 12, or 16 h. To inhibit MRE11 exo-/endonuclease ability, cells were cultured in the presence of either 25 μM Mirin (Fisher; 319010) or 25 μM PFM01 (Sigma; SML1735), respectively.

For fluorescent tagging of the pulse-labeled naDNA, the copper-catalyzed “Click” reaction was used as described in the Click-iT (ThermoFisher; C10640) protocol (75) with EdU contemporaneously with CPT damage. For visualization of ssDNA, cells were cultured with BrdU, which was detected using mouse monoclonal anti-BrdU (Abcam; 8039) without denaturation (24). Antibody labeling of proteins was achieved via direct and indirect labeling with Alexa Fluor 488 and 568 antibodies as detailed in *SI Appendix*, Table S3.

### SR Imaging and Analysis.

Prepared cells were imaged in the presence of an SR imaging buffer comprising an oxygen scavenging system (1 mg/mL glucose oxidase [Sigma-Aldrich; G2133], 0.02 mg/mL catalase [Sigma-Aldrich; C3155], and 10% glucose [Sigma-Aldrich; G8270] in phosphate-buffered saline) and 100 mM mercaptoethylamine (Fisher Scientific; BP2664100) (76). As described previously, all images were acquired on a custom-built SR microscope based on a Leica DMI 3000 inverted microscope (77) with 200 frames collected at 33 Hz for each color channel. Multicolor channels were corrected using a polynomial morph-type mapping algorithm before and a table of molecular localizations generated using the ImageJ (78) plugin QuickPALM (79). Rendered multicolor SR images were analyzed using three complementary approaches as detailed in Fig. 1 *C*–*E* and further discussed in *SI Appendix*, *Methods*. *N* values are in *SI Appendix*, Tables S1 and S2.

### Statistics.

Statistical analysis was carried out in OriginLab (8.5). All naDNA/protein overlap colocalization factors calculated were tested for significance against control samples (two-sample *t* test). All experiments were carried out in triplicate with total *N* sizes deemed acceptable. Unequal variances, particularly across temporal series, are expected.

## Supplementary Material

Supplementary File

## Data Availability

Data will be made available upon reasonable request via data transfer or hard drive shipment.

## Change History

March 18, 2021: The license for this article has been updated.
