# Supplementary material for: Super-resolution mapping of cellular double-strand break resection complexes during homologous recombination
Source: Proc Natl Acad Sci U S A. 2021 Mar 11;118(11):e2021963118. doi: 10.1073/pnas.2021963118 (PMC7980414; doi:10.1073/pnas.2021963118)
Supplement: Supplementary File [file pnas.2021963118.sapp.pdf]

SI Appendix Methods

Cell Culture and Drug Treatment

U2OS cells (ATCC HTB-96) were grown in McCoy's 5A (Modified) medium (ThermoFisher 16600) with 10% FBS (Gemini Bio. 100-106) and 100 U/mL Penicillin-Streptomycin (ThermoFisher 15140). Cells were seeded on glass coverslips and allowed to adhere in complete medium for 18-24 hours before being switched to FBS free medium for a further 48-72 hours in order to synchronize cells in G0/G1 phase. Cells were subsequently released in complete medium for a further 16 hours to produce a predominantly mid-S phase cell population(1). To induce RF stress and seDSB generation cells were then treated with 100 nM CPT (Abcam 120115) (2) alongside pulse labeling with 10 μM EdU, a thymidine analogue, for one hour so that it would be incorporated into naDNA during replication (Click-iT kit, ThermoFisher C10340)(3). Cells were fixed immediately following damage or released back into drug-free complete medium for a further 1, 2, 4, 8, 12 or 16 hours, which allowed for examination of the HR process over time. Control cells were treated with 0.1% DMSO in place of CPT.

To further perturb and probe the resection process of DSBs, U2OS cells were cultured in the presence of 25 μM of the MRE11 exonuclease inhibitor Mirin (Fisher, 319010) for 24 hours prior to recovery from CPT treatment (4). To do this, cells seeded on coverslips were administered Mirin during the final seven hours of serum starvation as well as during release in complete medium and in the CPT/EdU medium. Similarly, to inhibit endonuclease activity, cells were incubated in 25 μM PFM01 (Sigma, SML1735-5MG) 12 hours before CPT damage by addition to release medium in the final 12 hours. Following CPT treatment, cells were recovered for up to 16 hours in medium free of both CPT and Mirin/PFM01.

Extraction and Fixation

For visualization of chromatin and chromatin-bound nuclear fraction of cells, optimization of fixation and immunolabeling protocols was of paramount importance (5). In particular, the methods presented here were specifically optimized to achieve a high degree of soluble component extraction while maintaining the ultrastructure of the nucleus and are in good agreement with similar protocols (6, 7). Pre-extraction of the cells was achieved using room temperature CSK buffer (10 mM Hepes, 300 mM Sucrose, 100 mM NaCl, 3 mM $MgCl_2$, and 0.5% Triton X-100, pH = 7.4) for 2-3 minutes with gentle agitation. This was a key step as it removed the majority of the unbound fraction of HR proteins, which decreased the density of proteins within the nucleus and increased the proportion of detected proteins that were directly involved in HR (ie. associated with naDNA at DSB sites). This step also removed much of the cytosolic component, which minimized non-specific binding sites and sources of background and auto-fluorescence. After extraction, cells were fixed in paraformaldehyde (3.7% from 32% EM grade,

Electron Microscopy Sciences, 15714) and glutaraldehyde (0.3% from 70% EM grade, Sigma-Aldrich, G7776) in PBS for 15 minutes. The cells were then washed three times with PBS and, if required, stored overnight at 4°C. For fluorescent tagging of the pulse labeled nascent DNA, the copper catalyzed 'Click' reaction was used as described in the Click-iT (ThermoFisher, C10640) protocol(8). The cells were blocked with blocking buffer (2% glycine, 2% BSA, 0.2% gelatin, and 50 mM NH_4Cl in PBS) for 1 hour at room temperature (RT) or overnight at 4°C prior to further staining.

ssDNA and immunofluorescence labeling

For visualization of ssDNA, cells were cultured with BrdU either 11 hours prior to, and during, CPT treatment, or during CPT treatment only. No difference in overlap quantification was seen between approaches. After cell fixation and the naDNA 'Click' reaction, BrdU was then immunolabeled alongside protein immunolabeling with mouse monoclonal anti-BrdU (Abcam, 8039). This approach, without any denaturation of the DNA, has previously been demonstrated as limiting antibody access to, and fluorescent tagging of, the ssDNA(9).

Antibody labeling of all other proteins was achieved via a combination of direct and indirect labeling with Alexa Fluor 488 and 568 fluorophore labeled antibodies that have previously been validated in IF experiments. For a complete list see **SI Appendix Table 3.**

Super Resolution Imaging

For SR imaging, coverslips were stored for up to one week at 4°C before being mounted onto a microscope microfluidics chamber just prior to imaging. SR imaging buffer comprising an oxygen scavenging system (1 mg/mL glucose oxidase (SigmaAldrich, G2133), 0.02 mg/mL catalase (SigmaAldrich, C3155), and 10% glucose (SigmaAldrich, G8270) in PBS) and 100 mM mercaptoethylamine (Fisher Scientific, BP2664100) was then prepared fresh and added to the imaging chamber(10).

A custom-built SR microscope based on a Leica DMI 3000 inverted microscope was used to acquire all the raw data as has been described previously(11). Briefly, 473 nm (Opto Engine LLC, MBL-473-300 mW), 532 nm (OEM Laser Systems, MLL-III 200 mW) or 556 (UltraLasers, MGL-FN-556 200 mW) and 640 nm (OEM Laser Systems, MLL-III 150 mW) laser lines were combined using appropriate dichroics and focused onto the back aperture of a HCX PL APO 100X NA = 1.47 TIRF (Leica) objective via a multi-band dichroic (Chroma, zt405/488/532/640/730rpc, UF1C165837). The incident excitation beam could be translated laterally across the back of the objective to achieve a Highly Inclined and Laminated Optical (HILO) illumination configuration producing better signal-to-noise images.

Fluorescence emission was collected through the same objective and dichroic and imaged on an electron multiplying charge coupled device (EM-CCD) camera (Andor iXon+ 897). Fluorescence signal from AF568 and AF647 were collected simultaneously using a dual-band bandpass filter (Chroma, CY3/CY5, 59007m), and split into two channels on the EMCCD using a dichroic mirror (Semrock, FF660-Di02) in conjugation within a dual-view cube (Photometrics, DV2). AF488 signal was collected subsequently using a narrow single-band filter (Semrock, FF01-531/40). A 405 nm laser line (Applied Scientific Pro., SL-405 nm-300 mW) was introduced to enhance recovery of dark state fluorophores when required. S phase nuclei were identified by positive AF647 naDNA signal and 2000 frames at 33 Hz were acquired for each color.

A polynomial morph-type mapping algorithm achieved offset and chromatic correctional mapping of the three channels. To generate the correctional map, each day, prior to imaging, diffraction-limited images of spatially separated broad-range emitting fluorescent beads were acquired across all three colors (Tetraspecks, 100 nm, Life Technologies, T7279). The precise localizations of the beads were obtained by independently fitting their diffraction-limited Point Spread Functions (PSF) with Gaussian functions. The three-color sub-diffraction localizations could then be matched and an elastic mapping matrix generated based on the polynomial morph-type mapping function using an IDL (Exelis Visual Information Solutions) custom mapping script. Three-color raw image stacks of cellular images were then corrected before SR analysis.

Super Resolution Image Rendering and Analysis

To generate a table of molecular localizations and render SR images for colocalization analysis, the three acquired colors were processed independently using the ImageJ (12) plugin QuickPALM (13) with point spread function fitting constrained to spots with full-width half-maximum of 4 and signal-to-noise ratios better than 3. Images were rendered using 20 nm pixels and recombined to generate three-colour images over which masks were manually drawn identifying the nucleus based on the naDNA signal.

Using a Monte Carlo algorithm (14), 20 simulations of random rearrangements were generated for each nucleus in a pairwise fashion, examining Red/Green, Red/Blue, and Green/Blue overlap. The total number or area of overlaps detected in each nucleus (typically 15-100) could then be normalized to the determined random level of overlap expected by dividing the number/area of real overlaps by the average number/area of overlaps in the same simulated nucleus. An average normalized colocalization factor was thus calculated for each pairwise interaction between proteins, ssDNA, and naDNA (N typically 40-60 total cells, all N values available in **SI Appendix Table 1, 2**). A colocalization factor of

1 indicated completely random colocalization, while higher factors indicated association and interaction. Colocalization factors were also calculated for undamaged cells to establish control levels. Number of overlaps was used for 53BP1, Ku, MRE11, BRCA1, CtIP, and BLM, whereas area of overlap was used for H2AX, ssDNA and RPA, due to the expected accumulation of these proteins over multiple time points. In general the trends observed in number of overlaps were comparable to those observed in area but were more striking in the latter case for accumulating proteins.

To assess the prevalence of colocalization, dependence or exclusionary relationships between proteins at DSBs, all foci within a nucleus containing both naDNA and at least one of the two proteins stained were identified and quantified manually to determine the percentage of foci with colocalized proteins, as opposed to those positive only for one or the other. The average proportions were calculated and depicted in cumulative bar graphs.

To assess the intrafoci organization of proteins at DSBs, the distance between centers-of-mass of colocalized proteins at naDNA was determined. These distances were used to generate a histogram, which could be fit with a single of double Gaussian approximation. To standardize intrafoci two-color distances descriptive of closely associated proteins, RPA or RAD51 were double labeled with AF568 and AF488 and intrafoci distance histograms generated. In both cases, these histograms could be approximated with a Gaussian centered at an intrafoci distance of 135 nm with a width at half height of 70-80 nm. In cases where protein intrafoci distance histograms could similarly be fit with a Gaussian centered with a distance less than 180 nm, the relationship was considered intimate. In contrast, intrafoci distances which were approximated with a single Gaussian centered further than 180 nm described proteins occupying the same DSB but spatially separated. Histograms not easily described by a single Gaussian were well approximated by fitting one Gaussian at 135 nm distance and 75 nm full width at half height with free fitting of the total area of the Gaussian, and then free fitting a second Gaussian to describe the remaining intrafoci distances. Fitting two Gaussians described arrangements where in some foci the proteins were closely associated while in others they were spatially separated. To further visualize the spread of intrafoci distances observed, the fitted Gaussians were extrapolated into 3D contoured heat maps by fitting a second Gaussian perpendicular to the 2D Gaussian distance/intensity coordinates with the area of the perpendicular Gaussian proportional to the intensity value of the fitted Gaussians. These contoured heatmaps, shown throughout, in blue describe the likelihood of particular intrafoci distances being observed for different protein combinations at naDNA. The proximal-distal arrow included is consistently sized throughout the images and represents raw separation data values of 50 – 325 nm. We note that these values are not prescriptive of the underlying protein-protein or cluster-cluster separations, due to cumulative errors from mapping and localization

errors, steric labelling errors, and the blur and offset induced by labelling with multiple primary and secondary antibodies, which are, in turn, labelled with multiple fluorophores.

1. R. Khammanit, S. Chantakru, Y. Kitiyanant, J. Saikhun, Effect of serum starvation and chemical inhibitors on cell cycle synchronization of canine dermal fibroblasts. *Theriogenology* **70**, 27-34 (2008).
2. N. Saleh-Gohari *et al.*, Spontaneous homologous recombination is induced by collapsed replication forks that are caused by endogenous DNA single-strand breaks. *Molecular and Cellular Biology* **25**, 7158-7169 (2005).
3. Y. H. Chen *et al.*, ATR-mediated phosphorylation of FANCI regulates dormant origin firing in response to replication stress. *Molecular cell* **58**, 323-338 (2015).
4. A. Dupre *et al.*, A forward chemical genetic screen reveals an inhibitor of the Mre11-Rad50-Nbs1 complex. *Nature Chemical Biology* **4**, 119-125 (2008).
5. D. R. Whelan, T. D. M. Bell, Image artifacts in Single Molecule Localization Microscopy: why optimization of sample preparation protocols matters. *Scientific Reports* **5** (2015).
6. S. Britton, J. Coates, S. P. Jackson, A new method for high-resolution imaging of Ku foci to decipher mechanisms of DNA double-strand break repair. *Journal of Cell Biology* **202**, 579-595 (2013).
7. T. Aoto, N. Saitoh, Y. Sakamoto, S. Watanabe, M. Nakao, Polycomb group protein-associated chromatin is reproduced in post-mitotic G(1) phase and is required for S phase progression. *J. Biol. Chem.* **283**, 18905-18915 (2008).
8. M. Meldal, C. W. Tornoe, Cu-catalyzed azide-alkyne cycloaddition. *Chemical Reviews* **108**, 2952-3015 (2008).
9. E. Raderschall, E. I. Golub, T. Haaf, Nuclear foci of mammalian recombination proteins are located at single-stranded DNA regions formed after DNA damage. *Proc. Natl. Acad. Sci. U. S. A.* **96**, 1921-1926 (1999).
10. S. van de Linde *et al.*, Direct stochastic optical reconstruction microscopy with standard fluorescent probes. *Nature Protocols* **6**, 991-1009 (2011).
11. E. Agullo-Pascual *et al.*, Super-resolution fluorescence microscopy of the cardiac connexome reveals plakophilin-2 inside the connexin43 plaque. *Cardiovascular research* **100**, 231-240 (2013).
12. C. A. Schneider, W. S. Rasband, K. W. Eliceiri, NIH Image to ImageJ: 25 years of image analysis. *Nature Methods* **9**, 671-675 (2012).
13. R. Henriques *et al.*, QuickPALM: 3D real-time photoactivation nanoscopy image processing in ImageJ. *Nature Methods* **7**, 339-340 (2010).
14. K. Bermudez-Hernandez *et al.*, A Method for Quantifying Molecular Interactions Using Stochastic Modelling and Super-Resolution Microscopy. *Scientific Reports* **7**, 14882 (2017).

SI Appendix Table 1: N values for overlap analyses.

Drug Condition	Time	Species 1	Species 2	N
Control	0	naDNA	yH2A.X	30
CPT Only	12	naDNA	yH2A.X	55
CPT Only	16	naDNA	yH2A.X	36
CPT+Mirin	12	naDNA	yH2A.X	25
CPT+Mirin	16	naDNA	yH2A.X	55
Control	0	naDNA	53BP1	20
CPT Only	0	naDNA	53BP1	46
CPT Only	1	naDNA	53BP1	45
CPT Only	2	naDNA	53BP1	91
CPT Only	4	naDNA	53BP1	80
CPT Only	8	naDNA	53BP1	37
CPT Only	12	naDNA	53BP1	40
CPT Only	16	naDNA	53BP1	53
Control	0	naDNA	Ku	19
CPT Only	0	naDNA	Ku	103
CPT Only	1	naDNA	Ku	51
CPT+Mirin	0	naDNA	Ku	19
CPT+Mirin	1	naDNA	Ku	20
CPT+PFM01	0	naDNA	Ku	39
CPT+PFM01	1	naDNA	Ku	32
CPT+Mirin+PFM01	0	naDNA	Ku	27
CPT+Mirin+PFM01	1	naDNA	Ku	29
Control	0	naDNA	MRE11	37
CPT Only	0	naDNA	MRE11	96
CPT Only	1	naDNA	MRE11	48
CPT Only	2	naDNA	MRE11	104
CPT Only	4	naDNA	MRE11	76
CPT Only	8	naDNA	MRE11	37
CPT Only	12	naDNA	MRE11	31
CPT Only	16	naDNA	MRE11	52
CPT+Mirin	0	naDNA	MRE11	45
CPT+Mirin	1	naDNA	MRE11	47
CPT+Mirin	2	naDNA	MRE11	75
CPT+Mirin	4	naDNA	MRE11	68
CPT+PFM01	0	naDNA	MRE11	24
CPT+PFM01	1	naDNA	MRE11	30
CPT+PFM01	2	naDNA	MRE11	29
CPT+PFM01	4	naDNA	MRE11	29
CPT+Mirin+PFM01	0	naDNA	MRE11	28
CPT+Mirin+PFM01	1	naDNA	MRE11	22
CPT+Mirin+PFM01	2	naDNA	MRE11	18
CPT+Mirin+PFM01	4	naDNA	MRE11	16
Control	0	naDNA	CtIP	35
CPT Only	0	naDNA	CtIP	74
CPT Only	1	naDNA	CtIP	47
CPT Only	2	naDNA	CtIP	74
CPT Only	4	naDNA	CtIP	48
CPT Only	8	naDNA	CtIP	22
CPT Only	12	naDNA	CtIP	43
CPT Only	16	naDNA	CtIP	51
Control	0	naDNA	BRCA1	46
CPT Only	0	naDNA	BRCA1	81
CPT Only	1	naDNA	BRCA1	59
CPT Only	2	naDNA	BRCA1	68
CPT Only	4	naDNA	BRCA1	52
CPT Only	8	naDNA	BRCA1	36
CPT Only	12	naDNA	BRCA1	39
CPT Only	16	naDNA	BRCA1	47
CPT+Mirin	0	naDNA	BRCA1	19
CPT+Mirin	1	naDNA	BRCA1	23
CPT+Mirin	2	naDNA	BRCA1	26
CPT+Mirin	4	naDNA	BRCA1	19
CPT+PFM01	0	naDNA	BRCA1	19
CPT+PFM01	1	naDNA	BRCA1	27
CPT+PFM01	2	naDNA	BRCA1	16
CPT+PFM01	4	naDNA	BRCA1	19
CPT+Mirin+PFM01	0	naDNA	BRCA1	18
CPT+Mirin+PFM01	1	naDNA	BRCA1	26
CPT+Mirin+PFM01	2	naDNA	BRCA1	19
CPT+Mirin+PFM01	4	naDNA	BRCA1	19
Control	0	naDNA	EXO1	34
CPT Only	0	naDNA	EXO1	38
CPT Only	1	naDNA	EXO1	32
CPT Only	2	naDNA	EXO1	28
CPT Only	4	naDNA	EXO1	34

CPT Only	8	naDNA	EXO1	34
CPT Only	12	naDNA	EXO1	31
CPT Only	16	naDNA	EXO1	40
Control	0	naDNA	DNA2	25
CPT Only	0	naDNA	DNA2	27
CPT Only	1	naDNA	DNA2	29
CPT Only	2	naDNA	DNA2	26
CPT Only	4	naDNA	DNA2	28
CPT Only	8	naDNA	DNA2	29
CPT Only	12	naDNA	DNA2	25
CPT Only	16	naDNA	DNA2	22
Control	0	naDNA	BLM	26
CPT Only	0	naDNA	BLM	32
CPT Only	1	naDNA	BLM	47
CPT Only	2	naDNA	BLM	50
CPT Only	4	naDNA	BLM	53
CPT Only	8	naDNA	BLM	64
CPT Only	12	naDNA	BLM	33
CPT Only	16	naDNA	BLM	48
Control	0	naDNA	BrdU	16
CPT Only	0	naDNA	BrdU	52
CPT Only	1	naDNA	BrdU	62
CPT Only	2	naDNA	BrdU	48
CPT Only	4	naDNA	BrdU	77
CPT Only	8	naDNA	BrdU	33
CPT Only	12	naDNA	BrdU	40
CPT Only	16	naDNA	BrdU	54
Control	0	naDNA	RPA	63
CPT Only	0	naDNA	RPA	58
CPT Only	1	naDNA	RPA	62
CPT Only	2	naDNA	RPA	116
CPT Only	4	naDNA	RPA	68
CPT Only	8	naDNA	RPA	68
CPT Only	12	naDNA	RPA	66
CPT Only	16	naDNA	RPA	58
CPT+Mirin	0	naDNA	RPA	16
CPT+Mirin	1	naDNA	RPA	16
CPT+Mirin	2	naDNA	RPA	19
CPT+Mirin	4	naDNA	RPA	17
CPT+Mirin	8	naDNA	RPA	28
CPT+Mirin	12	naDNA	RPA	29
CPT+Mirin	16	naDNA	RPA	25

SI Appendix Table 2: N values for intrafoci analyses of WT+CPT damaged cells.

Time	Species 1	Species 2	N
0	MRE11	BRCA1	85
0	CtIP	BRCA1	83
0	MRE11	CtIP	87
0	53BP1	BRCA1	112
0	MRE11	BLM	113
1	MRE11	NBS1	117
1	MRE11	BRCA1	73
1	CtIP	BRCA1	65
1	MRE11	CtIP	87
1	53BP1	BRCA1	109
1	MRE11	BLM	129
1	EXO1	BLM	107
1	DNA2	BLM	98
1	EXO1	DNA2	124
2	MRE11	BRCA1	60
2	CtIP	BRCA1	84
2	MRE11	BLM	120
2	MRE11	CtIP	66

SI Appendix Table 3: Antibody List

Target	Species/Conjugate	Product Code	Manufacturer	Dilutions	Refs
53BP1	rabbit polyclonal	AB21083	Abcam	1:200/1:1000	1
BLM	mouse monoclonal	SC13584	Santa Cruz	1:500/1:2000	2
BRCA1	mouse monoclonal	SC6954	Santa Cruz	1:500/1:2000	3
BRCA1	mouse monoclonal AF488 conjugated	NB100-598AF488	Novus	1:250	*
BrdU	mouse monoclonal	AB8039	Abcam	1:200/1:500	4
CtIP	mouse monoclonal	SC271339	Santa Cruz	1:500/1:2000	5
DNA2	rabbit polyclonal	AB220883	Abcam	1:200/1:500	*
DNA2	mouse monoclonal	SC393323	Santa Cruz	1:200/1:500	6
EXO1	rabbit polyclonal	PA5-84141	ThermoFisher	1:500/1:2000	*
Ku	mouse monoclonal	ms-286	ThermoFisher	1:1000/1:5000	7
MRE11	mouse monoclonal	NB100-473AF488	Novus	1:200	8
MRE11	rabbit polyclonal	NB100-142	Novus	1:500/1:2000	9
NBS1	rabbit monoclonal	AB32074	Abcam	1:500/1:2000	10
RAD51	rabbit polyclonal	39194	Active Motif	1:500/1:1000	11
RAD51	mouse monoclonal	GTX70230	Genetex	1:500/1:2000	12
yH2A.X	rabbit polyclonal	NB100-384	Novus	1:2000/1:10000	13
	goat-anti-rabbit AF568	A11036	Invitrogen		
	goat-anti-rabbit AF488	A11034	Invitrogen		
	goat-anti-mouse AF568	A11031	Invitrogen		
	goat anti-mouse AF488	A11029	Invitrogen		

* denotes antibodies used which had not been used for IF applications in publications previously. To validate these antibodies they were double-stained alongside validated antibodies for the same target and found to have good colocalization.

1 Hernandez, L. *et al.* Increased Mammogram-Induced DNA Damage in Mammary Epithelial Cells Aged In Vitro. *Plos One* **8**, doi:10.1371/journal.pone.0063052 (2013).

2 Petsalaki, E., Dandoulaki, M., Morrice, N. & Zachos, G. Chk1 protects against chromatin bridges by constitutively phosphorylating BLM serine 502 to inhibit BLM degradation. *Journal of Cell Science* **127**, 3902-3908, doi:10.1242/jcs.155176 (2014).

3 Castella, M. *et al.* FANCI Regulates Recruitment of the FA Core Complex at Sites of DNA Damage Independently of FANCD2. *PLoS Genetics* **11**, doi:10.1371/journal.pgen.1005563 (2015).

4 Kranz, D., Dohmesen, C. & Dobbelstein, M. BRCA1 and Tip60 determine the cellular response to ultraviolet irradiation through distinct pathways. *Journal of Cell Biology* **182**, 197-213, doi:10.1083/jcb.200712014 (2008).

5 Huhn, D., Kousholt, A. N., Sorensen, C. S. & Sartori, A. A. miR-19, a component of the oncogenic miR-17 similar to 92 cluster, targets the DNA-end resection factor CtIP. *Oncogene* **34**, 3977-3984, doi:10.1038/onc.2014.329 (2015).

6 Xu, W., Wang, X., Chen, S., Wu, H., Tanaka, S., Onda, K., Sugiyama, K., Yamada, H. & Hirano, T., Tetrandrine enhances glucocorticoid receptor translocation possibly via inhibition of P-glycoprotein in daunorubicin-resistant human T lymphoblastoid leukemia cells. *Eur. J. Pharmacol.* **881**, doi: 10.1016/j.ejphar.2020.173232 (2020).

7 Reid, D. A. *et al.* Organization and dynamics of the nonhomologous end-joining machinery during DNA double-strand break repair. *Proc. Natl. Acad. Sci. U. S. A.* **112**, E2575-E2584, doi:10.1073/pnas.1420115112 (2015).

8 Uziel, T. *et al.* Requirement of the MRN complex for ATM activation by DNA damage. *Embo Journal* **22**, 5612-5621, doi:10.1093/emboj/cdg541 (2003).

9 Lee, K. Y. *et al.* MCM8-9 complex promotes resection of double-strand break ends by MRE11-RAD50-NBS1 complex. *Nature Communications* **6**, doi:10.1038/ncomms8744 (2015).

10 Yashavarddhan, M.H., Shukla, S.K., Chaudhary, P., Srivastava, N.N., Joshi, J., Suar, M. & Gupta, M.K. Targeting DNA Repair through Podophyllotoxin and Rutin Formulation in Hematopoietic Radioprotection: An in Silico, in Vitro, and in Vivo Study. *Front Pharmacol.* **8**, 750. doi:10.3389/fphar.2017.00750 (2017)

11 Bennett, B. T. & Knight, K. L. Cellular localization of human Rad51C and regulation of ubiquitin-mediated proteolysis of Rad51. *J. Cell. Biochem.* **96**, 1095-1109, doi:10.1002/jcb.20640 (2005).

12 Pfaffle, H. N. *et al.* EGFR-Activating Mutations Correlate with a Fanconi Anemia-like Cellular Phenotype That Includes PARP Inhibitor Sensitivity. *Cancer Res.* **73**, 6254-6263, doi:10.1158/0008-5472.can-13-0044 (2013).

13 Francia, S., Cabrini, M., Matti, V., Oldani, A. & di Fagagna, F. D. DICER, DROSHA and DNA damage response RNAs are necessary for the secondary recruitment of DNA damage response factors. *Journal of Cell Science* **129**, 1468-1476, doi:10.1242/jcs.182188 (2016).
